# Regulación del calcio por SERC-A antes de la enfermedad de Alzheimer y durante la misma

**DOI:** 10.7705/biomedica.6704

**Published:** 2023-03-30

**Authors:** Alamira Farah Alwiraikat-Flores, Pablo Octavio-Aguilar

**Affiliations:** 1 Laboratorio de Genética, Área Académica de Biología, Universidad Autónoma del Estado de Hidalgo, Hidalgo, México Universidad Autónoma del Estado de Hidalgo Universidad Autónoma del Estado de Hidalgo Hidalgo Mexico

**Keywords:** enfermedad de Alzheimer, ATPasas transportadoras de calcio, trastornos del metabolismo del calcio, receptor de N-metil-D-aspartato, retículo endoplásmico, Alzheimer disease, calcium-transporting ATPases, calcium metabolism disorders, N-methyl-D-aspartate receptor, endoplasmic reticulum

## Abstract

Hay muchos factores implicados en la incidencia de la enfermedad de Alzheimer que, en combinación, terminan por impedir o dificultar las funciones neuronales normales. Actualmente, poco se conoce sobre la regulación del calcio, antes de la enfermedad y durante la misma. La inestabilidad interna de los niveles de calcio se asocia a un mayor riesgo vascular, condición prevalente en un gran número de individuos ya comprometidos por la enfermedad de Alzheimer.

Esta revisión proporciona una reevaluación de los mecanismos moleculares de la ATPasa dependiente de Ca^2+^ del retículo sarcoendoplásmico (SERC-A) en la enfermedad y analiza los aspectos más destacados de la función de los canales de calcio dependientes de voltaje; de esta manera, se podrán abrir nuevas alternativas de tratamiento. Estos mecanismos de regulación son clínicamente relevantes, ya que se ha implicado la función irregular de SERC-A en diversas alteraciones de la función cerebral.

## Abstract (en)

There are many factors involved in the incidence of Alzheimer’s disease that, in combination, impede or hinder normal neuronal functions. Little is currently known about calcium regulation before and during the disease. Internal instability of calcium levels is associated with increased vascular risk, a prevalent condition in a high number of individuals already compromised by Alzheimer’s disease.

This review provides a reevaluation of the molecular mechanism of the sarcoendoplasmic reticulum calcium ATPase (SERC-A) in the disease and discusses salient aspects of voltage-gated calcium channel function; in these way new alternatives could be open for its treatment. These regulation mechanisms are clinically relevant since the irregular functions of SERC+A has been implicated in pathologies of brain function.

La enfermedad de Alzheimer es la causa más común de demencia humana, sobre todo en mayores de 60 años. Actualmente, más de 46 millones de personas en el mundo sufren de enfermedad de Alzheimer y se estima que para el 2050 este número aumente a más de 131 millones ^1^^,^^2^.

Esta enfermedad se debe a la pérdida progresiva de neuronas en diferentes partes del cerebro, lo que causa atrofia neurológica principalmente del hipocampo, estructuras corticales y límbicas, modificaciones patológicas que solo pueden ser evaluadas *post mortem*. Hay muchos factores implicados en la aparición de esta enfermedad, sobre todo si dificultan las funciones neuronales normales, como los accidentes vasculares, las condiciones de estrés prolongado o la falta de estímulos externos de refuerzo en el aprendizaje ^2^^-^^4^. También, están los factores ambientales que pueden provocar anomalías en el citoesqueleto (colchicina, genotoxicidad por plomo, etc.) o elementos endocrinos asociados con la expresión sexual, puesto que las mujeres presentan una mayor prevalencia ^5^^,^^6^; además de la edad, ya que aparece en el 50 % de los casos de personas mayores de 80 años y en el 15 % de aquellos entre los 65 y los 80 años ^7^.

Por lo tanto, la enfermedad de Alzheimer no es una entidad que se pueda explicar por un único evento anómalo, sino que es el resultado de la conjunción entre factores extrínsecos, como los ya mencionados, y alteraciones intrínsecas de la proteína β-amiloide (Αβ) (no en todos los casos), la acumulación de agregados proteicos sobre vainas de mielina o núcleos nerviosos, los procesos asociados con la cascada inflamatoria, el daño neuronal oxidativo por disfunción mitocondrial, las alteraciones proteicas de la molécula Tau, la formación de ovillos neurofibrilares, el fallo sináptico y el agotamiento de neurotransmisores, así como la herencia autosómica dominante del alelo 4 de la apolipoproteína E (APOEᵋ4) y mutaciones en las proteínas precursora amiloide (PPA) y presenilina-1 y 2; todo se ha correlacionado con casos de enfermedad de Alzheimer precoz familiar ^4^^,^^8^^,^^9^. En cualquier caso, la manifestación clínica de la enfermedad se asocia con una compleja progresión neurodegenerativa que produce un deterioro de la memoria y la pérdida de otros procesos cognitivos y no cognitivos ^10^.

En general, varios trastornos neurológicos, incluyendo Parkinson y Alzheimer, se vinculan por incidencia con alteraciones cardiovasculares, pero los elementos moleculares relacionados entre ambos procesos no se han delimitado apropiadamente, aunque se mencionan cambios osmóticos mediados por Ca^2+^ posteriores a las alteraciones mencionadas ^11^. De allí, que la inestabilidad celular de los niveles de calcio se asocie con un mayor riesgo vascular y su regulación abre una amplia gama de tratamientos para enfermedades renales, trasplantes y problemas cardiacos ^12^^-^^14^, aunque, actualmente poco se conoce sobre la regulación del calcio antes de la enfermedad de Alzheimer y durante la misma, y si se relaciona con la irrigación vascularización.

En este trabajo, se revisan los mecanismos moleculares de la regulación del Ca^2+^ durante la enfermedad de Alzheimer, con el fin de establecer si las ATPasas, específicamente la dependiente de Ca^2+^ del retículo sarcoendoplásmico (SERC-A), podrían ser un posible blanco terapéutico para el tratamiento.

## El calcio como regulador del potencial sináptico

La función neuronal parte de emitir y recibir señales que se propagan a través de la membrana celular por cambios en la permeabilidad plasmática. Como consecuencia, se desarrolla un potencial eléctrico que se propaga a lo largo de toda la neurona presináptica hasta la liberación de señales químicas en la hendidura o sinapsis, que permiten la transición de la señal hacia las terminales dendríticas de la neurona postsináptica donde se encuentran los receptores de tales señales ^15^.

Durante este proceso, la llegada de un potencial de acción al terminal presináptico induce la despolarización de la membrana en esa zona, lo que provoca que se abran los canales de Ca^2+^ dependientes de voltaje, receptores de N-metil-D-aspartato (NMDAR) y receptores de acetilcolina nicotínicos Alpha 7 (nAChR); generando que la concentración de Ca^2+^ citosólico de las neuronas en reposo, que oscila entre 50 a 300 μM, aumente hasta el orden de μΜ en la zona activa durante algunos microsegundos.

**Figura 1:**
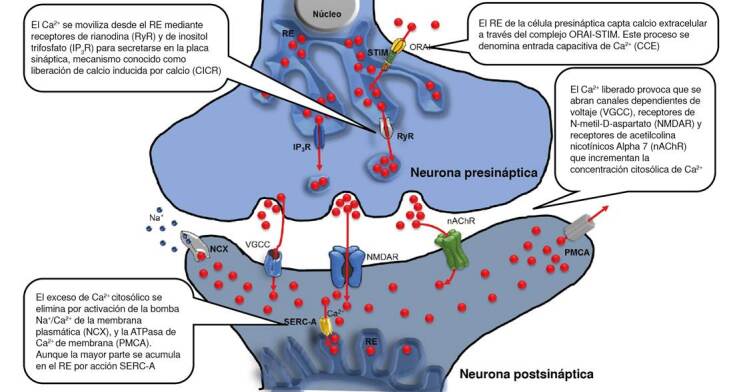
El calcio como regulador del potencial sináptico

Este incremento súbito es necesario para la sincronización en la liberación de neurotransmisores a la hendidura presináptica. Después, para recuperar el potencial de reposo, es necesario reducir el nivel de Ca^2+^ citosólico de nuevo, para lo cual se requiere la acción de bombas de Ca^2+^ como la bomba Na^+^/Ca^2+^ de la membrana plasmática (NCX) y la ATPasa de C^2+^ de la membrana plasmática (PMCA). Además, es posible secuestrar el exceso del ion en la luz del retículo endoplásmico mediante bombas que utilizan ATP para capturar el Ca^2+^ en estos depósitos intracelulares hasta alcanzar el potencial previo a la liberación (figura 1) ^16^^,^^17^.

Aun cuando las bombas de Ca^2+^ tienen una baja concentración en las células nerviosas, desempeñan un papel fundamental en su metabolismo y fisiología, controlando procesos que dependen de la amplitud, la frecuencia y la localización subcelular de las señales de Ca^2+ (^^17^^,^^18^, como la plasticidad neuronal, el impulso nervioso, el envejecimiento neuronal o la apoptosis ^19^. Numerosas enfermedades neurológicas, entre ellas la enfermedad de Alzheimer, llevan consigo una alteración de la homeostasis del Ca^2+^ o deficiencias en el funcionamiento de las bombas ^19^.

## ATPasas transportadoras de Ca^2+^ y su papel en las neuropatías degenerativas

La entrada capacitiva de Ca^2+^ es esencial para la homeostasis del Ca^2+^. Mantiene concentraciones adecuadas y funcionales en el retículo endoplásmico; así, este orgánulo es el principal reservorio de calcio celular, el cual posibilita una señalización sostenida por movilización del ion. La entrada capacitiva de Ca^2+^ se basa en un mecanismo de retroalimentación activado por la disminución del Ca^2+^ en el interior del retículo endoplásmico, la que desencadena su entrada a través de la membrana plasmática ^20^^-^^22^.

Las moléculas de interacción del estroma y el producto de expresión del gen *Orai1*, proteína estructural del canal iónico selectivo de calcio activado por la liberación de calcio 1, son los principales actores en la entrada capacitiva de Ca^2+^. Las moléculas de interacción del estroma detectan el contenido de Ca^2+^en el interior del retículo endoplásmico y, cuando disminuye, activa la transcripción de *Orai1*, lo que se traduce en un canal de calcio operado por depósitos intracelulares en la membrana plasmática. El destino final del Ca^2+^ que entra no es el citosol, sino el retículo endoplásmico, que se rellena muy eficientemente con él ^23^^,^^24^. La ATPasa de Ca^2+^ del retículo sarcoendoplásmico (SERC-A) es el tercer elemento de la entrada capacitiva de Ca^2+^, al que está estrechamente acoplado. La estrecha proximidad entre los depósitos intracelulares y la SERC-A favorece el rápido bombeo de Ca^2+^ desde los microdominios con abundante Ca^2+^ generados en la boca citoplasmática de los depósitos intracelulares hacia el retículo endoplásmico ^25^ (figura 1).

Las ATPasas transportadoras de Ca^2+^ presentan una gran afinidad y son responsables del transporte activo del ion a expensas de la hidrólisis de ATP en distintos tipos de membranas celulares. Se han identificado tres familias: la Ca^2+^-ATPasa de retículo sarcoendoplásmico (SERC-A), la Ca^2+^-ATPasa de membrana plasmática (PMCA) y la Ca^2+^-ATPasa de vías secretoras (SPCA), además de otros miembros de la familia especializados en el intercambio de iones H^+^/K^+^, Na^+^/K^+ (^^25^^,^^26^.

La SERC-A es una proteína anfifílica, integrada en las membranas del retículo sarcoendoplásmico que transporta dos iones Ca^2+^ desde el citoplasma a la luz de esos compartimentos, utilizando la energía de hidrólisis del ATP en presencia de Mg^2+ (^^26^. Esta proteína se identificó y purificó por primera vez en retículo sarcoplásmico de músculo esquelético, en donde se encuentra la isoforma SERC-A1; esta constituye el 90 % del total de proteínas de membrana y desempeña un papel muy importante en la contracción o relajación muscular, aunque con el tiempo se han descrito varias otras isoformas en diferentes tejidos ^27^.

**Figura 2:**
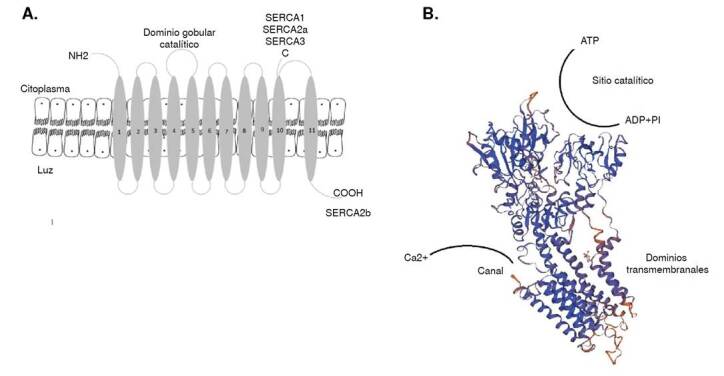
La Ca^2+^-ATPasa de retículo sarcoendoplásmico. A) Representación esquemática de la estructura de SERC-A, que muestra los segmentos transmembranales numerados, el dominio catalítico y los extremos N y C-terminal para las distintas isoformas. B) Modelo tridimensional de la SERC-A1.

La proteína está constituida por unos 1.000 aminoácidos con un peso molecular de 110 kDa. Presenta los extremos N-terminal y C-terminal hacia el citoplasma (SERC-A1,2a y 3); ocasionalmente, el C-terminal se orienta hacia la luz (SERC-A2b), tiene 10 (SERC-A1,2a y 3) a 11 (SERC-A 2b) dominios transmembranales. Estos dominios transmembranales que también conforman el canal de calcio, presentan una cabeza globular constituida por dos dominios citoplasmáticos, uno de los cuales es el dominio catalítico, donde se encuentra un residuo de ácido aspártico que se fosforila durante la activación y el sitio de unión del ATP (figura 2) (26-28).

Las isoformas 1, 2 y 3 se producen por tres genes muy conservados localizados en diferentes cromosomas que, además, generan diversidad adicional mediante el procesamiento alternativo de los ARN mensajeros a partir de tres dominios localizados en el extremo COOH-terminal ^26^. Hasta la fecha, se han identificado hasta 10 isoformas de SERC-A a nivel de proteína con gran especificidad por tejido y por etapa del desarrollo ^27^.

La isoforma SERC-A1 es específica de adultos, mientras que la forma B es propia de neonatos; ambas difieren en su región C-terminal ^28^. Son específicas del músculo esquelético de contracción rápida, donde contribuyen al flujo del Ca^2+^ implicado en la contracción o relajación muscular; la SERC-A1a conforma el 90 % de la proteína total en el músculo estriado. Estas variantes son codificadas por el gen *ATP2A1* localizado en la región cromosómica 16p12 ^29^.

Las mutaciones autosómicas recesivas en el gen *ATP2A1* se han asociado con la enfermedad de Brody en humanos ^30^, una miopatía rara y hereditaria caracterizada por un aumento perjudicial de la relajación del músculo esquelético durante el ejercicio, y que produce rigidez y calambres. Llama la atención que la ataxia asociada con la enfermedad de Alzheimer tenga una sintomatología similar.

La SERC-A2b es la principal isoforma del tejido nervioso ^31^, aunque también se localiza en el músculo liso y en tejidos no musculares como la piel. Las mutaciones autosómicas dominantes están asociadas con la enfermedad de Darier en humanos ^32^, una alteración de la piel caracterizada por la pérdida de adhesión entre las células epidérmicas y una queratinización anormal. En algunas familias con esta enfermedad, se han descrito, además, problemas neuropsiquiátricos como epilepsia, esquizofrenia, trastorno bipolar y depresión ^33^.

Llama la atención que los ARNm generados por recombinación alternativa de intrones para la isoforma SERC-A2a, son mucho menos estables que sus contrapartes del tejido muscular, por lo que se asume que el sistema de transporte del calcio es mucho más sensible en el tejido nervioso ^34^.

Por otro lado, la disminución en la expresión de este canal induce dolor neuropático. La inhibición del canal causa hiperexcitación neuronal, lesión nerviosa, estrés del retículo endoplásmico, activación de las células gliales satélite y alodinia mecánica (dolor debido a estímulos que normalmente no son dolorosos). Por lo anterior, los activadores de SERC-A2b tienen el potencial para el tratamiento del dolor neuropático.

Lo más importante de esta propuesta es que la sobreexpresión de SERC- A-2b después de lesiones por constricción crónica, produce un alivio a largo plazo de la alodinia mecánica y térmica, acompañado de restauración morfológica y funcional del tejido nervioso mediante el alivio del estrés del retículo endoplásmico ^35^; por ello, su uso en la enfermedad de Alzheimer permitiría la recuperación neurológica funcional, al menos parcialmente.

## Estrategia terapéutica para la enfermedad de Alzheimer basada en la regulación de SERC-A

La disminución de la concentración de Ca^2+^ en el retículo endoplásmico se ha establecido como una de las principales causas de la apoptosis inducida por el estrés del retículo endoplásmico ^36^. Aunque hay varios datos que muestran que la Aβ afecta a la homeostasis del Ca^2+^, los datos emergentes sugieren que los depósitos de Ca^2+^ del retículo endoplásmico están significativamente implicados en la producción de Aβ y en la fosforilación de tau durante la enfermedad de Alzheimer.

Infortunadamente, hasta el momento no hay cura para la enfermedad de Alzheimer. Los únicos tratamientos aprobados son los moduladores de neurotransmisores, que consisten en inhibidores de la colinesterasa, y el antagonista de los receptores de N-metil-D-aspartato, la memantina. Aunque estos tratamientos se dirigen a los síntomas de la enfermedad de Alzheimer y pueden proporcionar cierto alivio y comodidad a los pacientes, no detienen la progresión de la enfermedad en sí. La única característica que se ha correlacionado sistemáticamente con la progresión de la demencia es la pérdida de neuronas en los cerebros de los pacientes con la enfermedad de Alzheimer ^37^.

De hecho, los cerebros de las personas con enfermedad de Alzheimer muestran un recuento de neuronas tres veces menor en el hipocampo, en comparación con los cerebros de quienes no presentan esta alteración antes de los cincuenta años ^38^.

Por lo tanto, existe una clara necesidad de tratamientos que puedan dirigirse a este mecanismo de progresión de la enfermedad de Alzheimer. Recientemente, se ha informado sobre la capacidad del activador alostérico de SERC-A, CDN1163, para aliviar la acinesia parkinsoniana en ratas ^39^, y se reporta una eficacia convincente en el modelo de ratón transgénico para el precursor de la proteína amiloide y para la presenilina-1 (APP/PS1), con enfermedad de Alzheimer ^40^. Ambas proteínas se encuentran implicadas en la secreción del complejo gamma y la producción de Aβ en respuesta al estrés del retículo endoplásmico que, además, induce una reacción inflamatoria relacionada con la patogénesis de varias enfermedades ^41^. Recientemente, la administración de terapia génica a la SERC-A ha aliviado el estrés del retículo endoplásmico en varios modelos animales ^42^^,^^43^.

El mencionado estrés induce la modificación del plegamiento de varias proteínas que implica tanto reacciones inmediatas en los patrones de fosforilación celular, así como como cambios posteriores en la expresión de cientos de genes diana ^44^. El propósito de estos efectos adaptativos es restaurar la homeostasis celular o, al menos, intentarlo. Sin embargo, si el estrés provocado es prolongado, la modificación del plegamiento de varias proteínas puede desencadenar el programa de muerte celular apoptótica dentro de la célula que, en el caso de las neuronas, antecede incluso a la acumulación de Aβ y está directamente relacionado con la señalización proinflamatoria ^45^^,^^46^. En todo caso, la vía afectada se inicia con SERC-A y termina con dos vías de muerte neuronal.

El aumento de la actividad de la SERC-A mantiene el calcio del retículo endoplásmico y, por tanto, su función, a pesar de los factores de estrés. Además, la activación de la SERC-A puede secuestrar más Ca^2+^ citosólico y evitar la apoptosis inducida por la señalización mitocondrial. Todos estos factores apuntan a que la activación farmacológica de la SERC-A tendrá un impacto significativo en el tratamiento de la enfermedad de Alzheimer ^42^.

Esto ya ha sido probado en líneas celulares CSM14.1, obtenidas a partir de neuronas progenitoras estriatales de ratas previamente tratadas con tapsigargina, un inductor conocido de estrés del retículo endoplásmico que descarga los depósitos de la luz en el retículo endoplásmico por inhibición específica de SERC-A ^47^. Un pretratamiento con el compuesto de quinolina-amida CDN11163, fármaco inductor de la actividad de SERC-A, demostró la capacidad de rescatar las células del proceso apoptótico al reiniciar la función de la ATPasa.

Este resultado se ha obtenido también en células HEK, HeLa y BMGK expuestas a tapsigargina y peróxido de hidrógeno, como inductores de estrés del retículo endoplásmico cuando se usa CDN1163 ^47^^-^^49^. De igual manera, al proporcionar el fármaco a ratas mutantes de SERC-A, que en un principio aceleraron la pérdida de Ca^2+^ por excitotoxicidad inducida por glutamato (un neurotransmisor estimulante de los canales NMDA), se logró atenuar el estrés del retículo endoplásmico por agotamiento del ion ^50^. Por tanto, la estrategia terapéutica es clara: la activación de la SERC-A con CDN1163 o similares rellenará las reservas de Ca^2+^, aliviando el estrés del retículo endoplásmico, y rescatará eficazmente a las neuronas lesionadas de la apoptosis.

## Conclusión

El estrés ocasionado por una desregulación de los niveles de Ca^2+^ en el retículo endoplásmico de las neuronas, ocasiona su apoptosis, por lo cual es un factor determinante asociado con la enfermedad de Alzheimer. Por lo tanto, la estimulación de la ATPasa dependiente de Ca^2+^ del retículo sarcoendoplásmico (SERC-A), podría ser un posible blanco terapéutico en dicha enfermedad, al reducir los niveles de Ca^2+^ en el citosol de las neuronas del hipocampo.
